# Prognostic variables and scores identifying the end of life in COPD: a systematic review

**DOI:** 10.2147/COPD.S137868

**Published:** 2017-07-31

**Authors:** Laura-Jane E Smith, Elizabeth Moore, Ifrah Ali, Liam Smeeth, Patrick Stone, Jennifer K Quint

**Affiliations:** 1 Department of Respiratory Epidemiology, Occupational Medicine and Public Health, Imperial College London; 2 Department of Epidemiology and Population Health, London School of Hygiene and Tropical Medicine, London; 3 Marie Curie Palliative Care Research Unit, University College London, UK

**Keywords:** COPD, palliative care, end of life

## Abstract

**Introduction:**

COPD is a major cause of mortality, and the unpredictable trajectory of the disease can bring challenges to end-of-life care. We aimed to investigate known prognostic variables and scores that predict prognosis in COPD in a systematic literature review, specifically including variables that contribute to risk assessment of patients for death within 12 months.

**Methods:**

We conducted a systematic review on prognostic variables, multivariate score or models for COPD. Ovid MEDLINE, EMBASE, the Cochrane database, Cochrane CENTRAL, DARE and CINAHL were searched up to May 1, 2016.

**Results:**

A total of 5,276 abstracts were screened, leading to 516 full-text reviews, and 10 met the inclusion criteria. No multivariable indices were developed with the specific aim of predicting all-cause mortality in stable COPD within 12 months. Only nine indices were identified from four studies, which had been validated for this time period. Tools developed using expert knowledge were also identified, including the Gold Standards Framework Prognostic Indicator Guidance, the RADboud Indicators of Palliative Care Needs, the Supportive and Palliative Care Indicators Tool and the Necesidades Paliativas program tool.

**Conclusion:**

A number of variables contributing to the prediction of all-cause mortality in COPD were identified. However, there are very few studies that are designed to assess, or report, the prediction of mortality at or less than 12 months. The quality of evidence remains low, such that no single variable or multivariable score can currently be recommended.

## Introduction

COPD is a major cause of morbidity and mortality and an important public health challenge.^1^–^3^ Prevalence is increasing globally, and according to projections COPD not only is the third leading cause of death but also will be the seventh leading cause of disability-adjusted life years (DALYs) lost worldwide by 2030.^4^

Systematic identification of patients approaching the “end of life” is a key recommendation of the end-of-life care strategy.^5^ The unpredictable disease trajectory of COPD^6^ makes this difficult, compounded by the fact that there is no “gold standard” method for predicting prognosis in COPD and no clear guidance on how to identify factors that may assist prognostication in the last year of life. Easily measurable physiological parameters and traditional measures of disease severity, such as forced expiratory volume in 1 second (FEV1) or Global Initiative for Chronic Obstructive Lung Disease (GOLD) stage 4 (a severity of airflow obstruction [FEV1 less than 30% predicted]), do not correlate well with mortality in individuals.^7^,^8^ There are growing calls from patients, health care professionals and policy makers for better tools for prognostication,^9^ particularly since clinicians’ predictions of survival are often inaccurate.^10^,^11^ Improvement in accuracy of prognostic tools has been identified as a research priority.^12^ Although there are debates as to whether prognosis is the best way to identify patients who should be offered a palliative care approach, systematic identification of those likely to be nearing the end of life could form an important part of a strategy to close the gap between need and receipt of a palliative approach to care,^13^–^17^ and may be an important way to help clinicians overcome “prognostic paralysis.”^18^

Survival time, advance care planning and patient–provider communication are among some of the 12 key variables proposed to determine the quality of end-of-life care.^19^ Information needs are noted to be frequently unmet for patients with COPD, their families and carers. Prognostic information could support shared decision-making, aid estimation of health care utilization and identify groups who would benefit from specific interventions as recommended in national and international guidelines.^20^,^21^

A number of variables have been identified which are useful in making predictions about prognosis in COPD and scores that combine a number of variables have been developed, in recognition of the fact that COPD is a multisystem disease.^22^ However, many of these have been developed to estimate long-term prognosis over many years. Prognostic factors that are useful in predicting 10- or 5-year prognosis may not be the most relevant factors to predict which patients are at greatest risk of death within the next year. Since policy literature consistently states that the last year of life is the time during which proactive identification of patients should occur, it is important to understand what tools and variables may aid clinicians in prognostic prediction over this time period.

Therefore, our objective was to investigate known prognostic variables and scores that predict prognosis in COPD in a systematic literature review, specifically including variables that contribute to risk assessment of patients for death within 12 months.

## Methods

### Study design

This was a systematic review. We aimed to investigate known prognostic variables and scores that predict prognosis in COPD, specifically those variables that contribute to risk assessment of patients in the community (ie, not hospitalized) for death within 12 months. We sought in particular to identify variables and tools that could be used in primary care at an “annual review” or stable COPD visit. No ethical approval was required, since this study is a synthesis of published studies.

The details of the protocol including the search terms used and all inclusion and exclusion criteria have been registered and published^23^ and can be found at www.crd.york.ac.uk/PROS-PERO (registration number CRD42016033866). We included studies of adults ≥35 years old with stable COPD with the outcome of interest of all-cause mortality. We excluded studies of patients with alpha-1-antitrypsin deficiency, or those who had undergone lung transplantation, lung volume reduction surgery or comparative interventional bronchoscopic procedures; studies in which COPD was a covariate, or in which people with COPD formed a subgroup and no separate reporting was available; studies in which prognostic variables were recorded at the time of an exacerbation or hospitalization (as they may not be relevant at a stable visit); studies that investigated prognostic markers not typically available in routine clinical care (eg, biomarkers in development or invasive investigations) and studies in which the only exposure was occupational or environmental (eg, air pollution).

We searched Ovid MEDLINE, EMBASE, the Cochrane database of systematic reviews, Cochrane CENTRAL, DARE and CINAHL up to December 30, 2015, and updated the search on May 1, 2016. We used medical subject heading and text words related to COPD, and broad strategies to identify prognostic studies and prognostic markers, focused on advanced disease and the end of life. We supplemented our search from other sources including reference lists of included studies, index-related articles on PubMed, and existing relevant reviews, as well as Google Scholar and ProQuest. For any prognostic indices identified, we performed forward and backward citation tracking to identify derivation and validation studies.^24^ The search strategy with selected terms was described in the previously published protocol.^23^

### Selection of studies and extraction of data

Prognostic studies are more challenging to identify in the literature than diagnostic or treatment studies, so a broad search strategy was used. There was often insufficient information in the abstract to determine whether the study was appropriate for inclusion and a large number of full texts were screened. Two authors screened the titles and abstracts of all literature retrieved by the initial search against inclusion and exclusion criteria and selected articles for full-text review. All data were downloaded to Zotero^25^ for data management. Two authors reviewed all full-text articles. Differences of opinion were resolved by consensus or by arbitration by a third reviewer. Two reviewers extracted data independently using a prespecified data extraction tool, including details of the study setting, study design, population, diagnostic criteria for COPD, method of measurement of each prognostic variable, outcome definition and funding source. The tool also included fields relevant to multivariate models based on the Critical Appraisal and Data Extraction for Systematic Reviews of Prediction Modelling Studies (CHARMS) checklist^26^ such as modeling method, handling of predictors, method for selection of predictors, shrinkage of predictor weights, univariate and multivariate associations, model performance and evaluation. This was piloted on the first five full-text reviews to ensure standardized use of the tool. A Preferred Reporting Items for Systematic Review and Meta-analysis Protocols (PRISMA-P)^27^ flow diagram was constructed.

### Quality assessment

Two reviewers assessed the quality and risk of bias of eligible studies based on prespecified domains. An approach based on the Quality in Prognostic Studies (QUIPS) tool,^28^ specifically designed for prognostic reviews, was used. We considered questions under six domains: study participation and attrition, prognostic factor measurement, outcome measurement, confounding measurement and account, analysis and reporting, and others.

### Data synthesis

A narrative synthesis of all identified evidence was completed. We summarized the range of outcome predictors that have been studied to date for the outcome of all-cause mortality within 12 months in COPD. Hazard ratios and odds ratios were extracted with 95% confidence interval (CI) where possible but were often not reported. With regard to composite scores, we assessed the quality of model building, the methods used to internally and externally validate the score and to what degree clinical utility and impact had been assessed. C-statistics with 95% CIs and measures of calibration including calibration plots and the Hosmer–Lemeshow test were extracted where possible.

We planned to assess the strength of evidence for each prognostic variable or score included based on Grading of Recommendations Assessment, Development and Evaluation (GRADE) evidence profiles.^29^ However, since the studies were so heterogeneous and there was such a paucity of evidence for any single variable or score, this was not possible.

## Results

**Figure 1:**
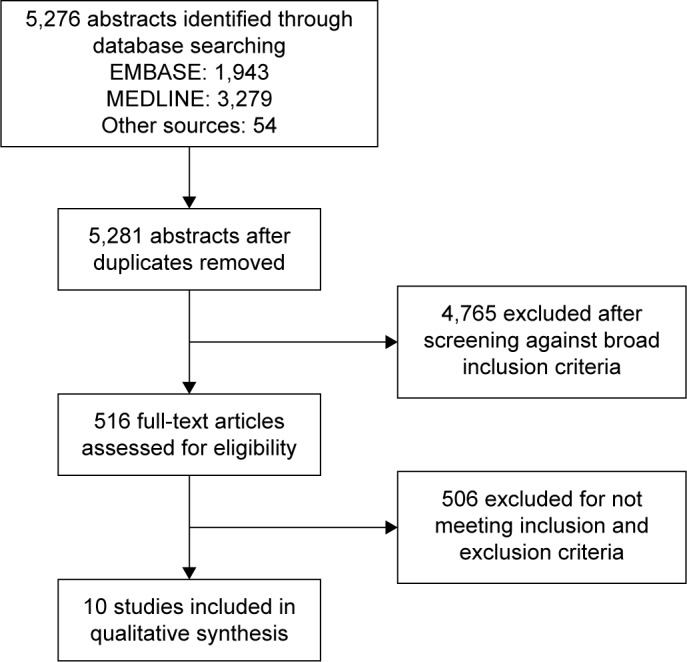
Screening process.

A total of 5,276 abstracts were screened, leading to 516 full-text reviews. Despite these large numbers, only 10 met full inclusion and exclusion criteria (PRISMA diagram, Figure 1). A large number of studies were excluded as they were conducted in hospitalized patients, or in subsets of patients with COPD such as those who had undergone surgery or who were on long-term oxygen and were therefore not representative of patients in the community with stable COPD. A significant number of studies included patients with COPD but did not report any associations between variables and mortality separately so could not be included. A large number of studies were excluded as, although they investigated prognostic variables or scores in COPD, they did not report outcomes ≤12 months. Table 1 describes the studies that investigated individual and multivariable prognostic variables for the prediction of mortality in stable COPD ≤12 months. Tables 2 and 3, respectively, describe the individual and multivariable prognostic indices predictive of mortality ≤12 months.

### Multivariable indices and scores predicting all-cause mortality ≤12 months

No studies reported multivariable indices developed with the specific aim of predicting all-cause mortality in people with stable COPD within 12 months of death. Only nine indices (six of which were truly multivariable, and three of which were comorbidity indices) were identified that had been used for this 1-year time period. Only in the case of the B-AE-D score (the four parameters being body mass index [BMI] [B], severe acute exacerbation of COPD [AECOPD] frequency [AE], modified Medical Research Council [mMRC] dyspnea severity [D]) was the study from which the score was derived identified. In all other cases, the studies presenting outcome at ≤12 months were not the original derivation of that predictive score, but rather validation studies.

Marin et al^30^ provided the most information, validating a number of existing prognostic indices in a large individual pooled dataset (n=3,633) from multiple cohort studies with different stages of COPD. All-cause mortality prediction at both 6 and 12 months was assessed (as well as 3, 5 and 10 years, not presented in this article). These included the original BODE index (BMI, airflow Obstruction, Dyspnea and Exercise) and three of its modifications (modified BODE [mBODE], BODE exercise capacity [x] and exacerbations BODE [eBODE]), the SAFE (obstruction, exercise, quality of life and exacerbations), ADO (age, dyspnea and obstruction) and DOSE (dyspnea, obstruction, smoking and exacerbation) indices (Table 4). These were compared to FEV1. Since the indices share construct variables, they were highly autocorrelated, and unsurprisingly there was little difference in discrimination between similar scores. In this study, the C-statistic for FEV1% predicted at 6 months was 0.657. Over this time period, ADO (C-statistic 0.7015), BODE (C-statistic 0.6808) and eBODE (C-statistic 0.6808) were the best to predict mortality, with DOSE score performing worst (C-statistic 0.632). At 12 months, the C-statistic for FEV1% predicted was 0.656, with ADO the best of the indices tested (C-statistic 0.701). Again DOSE score had the worst discrimination over this time period (C-statistic 0.631). In the Supplementary materials and Tables S1–S4, it was shown that, when adjusted for age, all BODE modifications showed superiority over both BODE and ADO but C-statistics were not individually reported. No CIs on C-statistics were presented, and there was no assessment of calibration, either by the preferred method of presentation of calibration plots or by calculation of the Hosmer–Lemeshow statistic.

In the study in which the newest index, the B-AE-D index, was developed, a number of existing indices were also tested for the prediction of all-cause mortality at 12 months, including ADO, BODE and the updated BODE score.

The study by Austin et al^31^ was quite different from the others, using Canadian administrative data to test different comorbidity classification schemes for predicting all-cause mortality at 12 months. All were shown to have good discrimination and calibration in both incident and prevalent populations, suggesting that comorbidity scores are useful in this context. However, without presentation of a sum score or the β coefficients of the regression models, it is not possible to replicate the methods or determine how different comorbidities were weighted, and this finding is therefore of limited application in practice, particularly outside the Canadian health care system.

In addition to those multivariable tools derived using statistical methods as discussed earlier, tools developed using expert knowledge were also identified. These draw together not only knowledge from existing studies but also a wealth of clinical experience. A major limitation is that, although a stated aim of several of these tools is to identify those approaching death, the success of these tools in achieving this aim has not been tested. Four tools were identified which fall into this category for COPD (details of the components of each tool are available in the Supplementary materials and Tables S1–S4): the Gold Standards Framework Prognostic Indicator Guidance (GSF-PIG),^32^ the RADboud Indicators of Palliative Care Needs (RADPAC),^33^ the Supportive and Palliative Care Indicators Tool (SPICT)^34^ and the Necesidades Paliativas (NECPAL) program tool.^35^

Table 4 describes the shared variables across multivariable indices predicting mortality. The fact that many indicators are shared among these tools provides evidence of a degree of international consensus about those factors of use in identifying those at risk of death who would benefit from palliative care. However, without evidence of reliable prognostic prediction, or other outcomes such as enhanced access to palliative care, it is not possible to confidently recommend any of these tools for use in practice. It is also not possible to compare them with other multivariable tools, developed using statistical methods. It was therefore not possible to include them in the review (further details of tools are given in the Supplementary materials and Tables S1–S4).

### Individual variables predicting ≤1 year mortality

There was very little evidence for any individual factor in predicting mortality, except in very selected populations (eg, National Emphysema Treatment Trial [NETT]),^36^ but in this study there was limited reporting for an outcome of 1-year mortality.

### Impact

No studies were identified as part of the review which assessed the clinical impact of the use of any variable or prognostic index for the identification of patients nearing the end of life. Guidelines on the management of COPD from the British Thoracic Society, European Respiratory Society, American Thoracic Society, Scottish Intercollegiate Guideline Network and GOLD guidelines were also searched to assess the impact of the multivariable scores identified in this review.

The UK National Institute for Health and Care Excellence (NICE) guidelines for COPD recommend that disability in COPD can be poorly reflected by FEV1 alone, and that a more comprehensive assessment includes other known prognostic factors (gas transfer for carbon monoxide [TLCO], breathlessness on Medical Research Council [MRC] scale, health status, exercise capacity, BMI, partial pressure of oxygen in arterial blood [PaO_2_] and cor pulmonale) and that the BODE index should be calculated to assess prognosis where its component information is available. These guidelines acknowledge that the additional time and cost of routinely performing 6-minute walk test (6MWT) in all patients may not be justified, particularly in a primary care setting. The BODE index is a routine part of the assessment criteria for lung transplantation for COPD in the UK.

The GOLD guidelines recommend the use of multidimensional prognostic indices, but do not specify which to use, in which circumstances, or settings. GOLD 2011^37^ suggests BODE where 6MWT is available, and BODEx when 6MWT is not available. The Spanish COPD guidelines recommend the use of BODEx for prognostic prediction in COPD, but not for the identification of those in the last year of life specifically.^38^

### Risk of bias

We considered possible spectrum bias^39^ and the implications for generalizability of our findings. These findings are summarized in Table 5. Overall, the studies were found to have a moderate risk of bias. The main risk derived from study participants who were not always representative of the general COPD population.

## Discussion

This review provides an important summary of what we know about the robustness of available tools to identify those in the last year of life with COPD and shows that more work is needed. A number of variables contributing to the prediction of all-cause mortality in COPD have been identified. Many of the same predictors are combined in different ways in different multivariable scores, which implies a degree of consensus among investigators as to the important factors. However, there are very few studies that are designed to assess, or which report, the prediction of mortality at or less than 12 months. The quality of evidence remains low, such that no single variable or multivariable score can currently be recommended.

The future brings great challenges for providing high-quality care for the growing population of patients living and dying with COPD, many of whom are cared for in the community. Unless systematic screening of patients with advanced respiratory disease becomes routine, many patients will not realize the benefits of identifying those nearing the end of life.^40^

Predicting death is a challenge not just for COPD but other conditions such as cardiovascular diseases with predictive models of cardiovascular disease providing similar C-statistics.^41^ Clinician predictions of prognosis are notoriously inaccurate but prognostication is a core clinical skill that must be both taught better, and supported with better evidence.^42^ There may be reluctance to prognosticate in the absence of an obvious treatment that can be offered. However, there is growing evidence that palliative care interventions improve a number of outcomes in both cancer and non-cancer diagnoses and have even shown a mortality benefit in a randomized controlled trial (RCT) in those with metastatic non-small cell lung cancer.^43^ Advance care planning and holistic palliative care programs with elderly patients, and those with a range of chronic diseases, have shown improvements in concordance with preferred place of death,^44^ symptom burden, quality of life and reduced distress and depression in surviving relatives.^45^ These studies began by identifying a population of patients at risk of death, selected due to age, general measures of frailty or dependence or disease-specific measures of severity. We need a robust starting point for such studies in COPD, but as this review shows we do not currently have one.

### Methodological limitations

A major limitation is that even those studies that met inclusion criteria were in selected populations, such as those with severe disease in the NETT RCT,^46^ or those with no significant comorbidities.^47^ They are not representative of the general COPD population, and it is therefore difficult to generalize any conclusions.

In addition, many prognostic indices have been derived in small groups of patients (Supplementary materials and Tables S1–S4). Methods which would maximize power, such as bootstrapping for internal validation as an alternative to splitting samples into derivation and validation samples, are underused. There was almost no mention of shrinkage or penalized regression methods in the derivation of the identified studies, leading to a high risk of overoptimism, particularly where there are few events in the context of a large number of potential predictors.^48^ This is demonstrated when indices which appeared to perform well in the original study, did not perform well when tested in other populations. Reporting of prognostic studies is of poor quality, making risk of bias assessments challenging. There is a lack of reporting on blinding of variables for each other, on the degree of missing data, on whether model assumptions are met particularly for continuous variables (eg, linear trends), and on methods of predictor selection. Again, this limits confidence in any of the prognostic scores.

A further problem is that prognostic studies have been conducted with many different aims and objectives. A variable or index developed to predict future hospitalization may not be optimal to predict mortality. Similarly, an index developed to predict mortality over a 5- or 3-year period may not perform well when used to attempt to predict 1-year mortality.^46^ In view of the fact that COPD is a heterogeneous multisystem disorder, we have perhaps expected too much from any one variable or index. From a methodological point of view, there is no reason to expect a prognostic score developed in one population for one purpose to perform well when applied to another population over a different time period. In contrast to calls for “no more risk scores,” the academic or clinical community perhaps needs to reach a consensus on which indices should be adopted systematically into practice for which purposes, and which questions are as yet unanswered by available tools. The discussion as to whether age should be included in prognostic indices is also relevant here, since if the aim is to derive a prognostic score that can act as a biomarker measured pre and post an intervention we would want it to be sensitive to change in disease-specific factors. However, if the aim is to precisely predict the risk of death for an individual over a relatively short time, then age is likely to be highly relevant. Those prognostic indices that have undergone external validation in various populations, such as BODE, BODEx and ADO, are likely to be of ongoing use in risk stratification. But to identify those likely to be in the last year of life, current tools are inadequate. Of note, no identified tools were developed in a UK population. Of course, all tools must be set within a clinical context in which clinician experience and patient preferences and needs are also integrated.

We recognize that one of the reasons it may be difficult to predict risk of death at 1 year in people with COPD is the fact that they die for multiple different reasons, not just their COPD. Use in clinical practice of scores that predict specific causes of death (eg, cardiovascular risk scores) may ultimately be more useful in certain subgroups of patients but was not the focus of this review.

### Future directions

To move forward, for the benefit of patients with COPD, we must continue to build on available evidence, using robust methods for prognostic model development, validation and updating. Although small individual cohort studies may provide data on individual or novel prognostic variables, to develop and validate multivariable tools for use in practice, we must move to the use of large datasets. In addition to pooled individual patient data from trials and observational cohort studies,^30^,^49^ we should seek to use other underexploited data such as electronic health record databases, particularly as they provide a means of developing and validating prognostic indices in populations of “real-world” patients, and are an efficient and cost-effective use of resources. An optimal risk prediction score to identify community COPD patients at high risk of death within the next year should be simple to use and use readily available risk factors, ideally those that are routinely captured in existing health records. This may include variables identified from existing models such as: FEV1% predicted, age, breathlessness and exacerbation frequency, but may also include other factors identified by consensus by experts, such as comorbidities, use of long-term oxygen therapy (LTOT), prolonged use of oral steroids and measures of frailty. Incorporation of the COPD specific comorbidity test score^50^ may improve existing indices. A larger number of predictors may be needed, to improve precision in risk estimates. With the use of online calculators, this is feasible. Large datasets also facilitate the development of tools which estimate individualized risk prediction, rather than placing patients into broad risk groups. Individualized risk prediction may better support clinical decision-making and shared decision-making.

Prognostic uncertainty and an unpredictable disease trajectory are features in common between advanced COPD and heart disease, but prognostic risk scores are already in widespread use in the cardiology community, in contrast to respiratory medicine. The EFFECT score provides an estimate of 30-day and 1-year mortality for those presenting to hospital with heart failure. A recently published Phase II RCT used this tool along with the GRACE score to define a 20% 12-month mortality risk as entry criteria for a trial of future care planning.^51^,^52^ This trial found that using such a risk-threshold approach was valid as a means of identifying a population of patients at high risk of death or deterioration, with a high burden of comorbidity who may therefore benefit from additional holistic or palliative care. This was despite concerns raised that prognostic estimates should not be the only route to palliative care interventions, and that needs-based assessment^53^ is also key, particularly for those with difficult to treat symptoms such as breathlessness. Such pathways should work in parallel, with better tools to aid prognostication supporting systematic identification of those who may benefit, particularly from advance care planning^54^,^55^ and other routes to palliative care services receiving equal support and attention.

## Conclusion

Current evidence does not allow clinicians to reliably predict which patients with COPD are approaching end of life, limiting ability to provide palliative care services appropriately.

## Supplementary materials

### Tools for the identification of patients in the last year of life in COPD which were identified as part of the review, but which have not been tested for accuracy

1. Gold Standards Framework Prognostic Indicator Guidance (GSF-PIG): The Gold Standards Framework is “a systematic, evidence based approach to optimising care for all patients approaching the end of life, delivered by generalist frontline care providers.” One part of the program is the provision of prognostic indicator guidance (GSF-PIG) which aims to identify those in the last year of life, to include them on the palliative care register, as when this is achieved “there is good evidence that they are more likely to receive well-coordinated, high quality care.”^1^ It is emphasized in the guidance that prognostication is inherently difficult, and that the focus should be on identification of needs, and “rainy-day thinking” to plan ahead for those at risk of decline and death. Any tool must be placed within a clinical context, used alongside clinical judgment rather than in place of it. However, any tools claiming to aid the identification of those in the last year of life should be assessed for accuracy and impact. Studies which include patients with COPD have been conducted in hospitalized patients assessing the predictive value of the GSF-PIG, suggesting that screening with GSF-PIG may be useful in this population, although based on very small numbers in a single center. No similar studies were identified in patients with COPD in the community.
2. RADboud Indicators of Palliative Care Needs (RAD-PAC): The RADPAC study^2^ proposed guidance on the identification of patients with COPD, heart failure and cancer nearing the end of life, developed through a literature review, focus group interviews and a modified Rand Delphi method. The literature review mainly identified prognostic indicators, while the focus groups included triggers to consider palliative care not necessarily related to prognosis. At the end of this process, six indicators were identified for COPD, to help general practitioners (GPs) identify patients in need of palliative care. RADPAC is under study in a randomized controlled trial including 158 GPs in the Netherlands, comparing the intervention to usual care. Outcomes will include quality of life, hospitalizations and other planned care, place of death and time before death that identification of palliative needs occurred.^3^
3. Supportive and Palliative Care Indicators Tool (SPICT): The SPICT was initially developed in 2010 by expert consensus as a guide to identify those at risk of deteriorating and dying who may benefit from supportive and palliative care. It was refined using a mixed-method approach, including peer review of multiple iterations of the tool via a web-based system, and a prospective case-finding study of patients with advanced renal, liver, cardiac or respiratory disease following an unplanned admission to an acute hospital followed up for 12 months. Although identified at hospital admission for this arm of the study, the prognostic indicators were designed to be used in both primary and secondary care. The indicators are not specific to COPD, but are for respiratory disease in general. Limited data are presented, but 17 patients with COPD were identified by the tool, 50% of whom had died by 12 months of follow-up.^1^ Interestingly in the earlier version of the tool, parameters were more specifically defined, and the tool included the surprise question, while in the later version parameters are broader and the surprise question has been removed.
4. Necesidades Paliativas (NECPAL) program: The NEC-PAL program is part of the World Health Organization (WHO) Demonstration Project on Palliative Care in Catalonia (Spain), aiming to improve palliative care in the region. It focuses on early identification and improved care of patients with advanced chronic conditions in the community. The NECPAL CCOMS-ICO tool^4^ has been developed as part of the program, aiming to predict 12-month risk of death for patients with chronic advanced diseases. It was based on the GSF-PIG and SPICT tools with additional indicators felt to be relevant to a Spanish health care setting added. The tool was evaluated by a multidisciplinary expert panel, and after five iterations a final tool was proposed. The tool has been used in a cross-sectional, population-based study to investigate the prevalence and characteristics of patients with advanced chronic conditions (including COPD) in need of palliative care, estimating that this was 1.5% of the population. An analysis of the tool’s predictive capacity for 12-month risk of death (Part III of study) has not yet been published.

**Table S1:** GSF-PIG^5^

Components of score	Means of classifying patients on the basis of score
The surprise question	No specific advice is given on the number of indicators or weighting of indicators which relate to stage of disease, other than to state that at least two disease-specific indicators should be identified. The guidance encourages “needs-based coding” to help identify those who should be on the palliative care register, and proactively plan care. A: stable = years. B: unstable advanced disease = months. C: deteriorating with exacerbations = weeks. D: last days of life = days.
General indicators • Decreased activity (Barthel index or in bed/chair >50%) and increasing dependence for ADLs • Comorbidities • Deteriorating complex symptom burden • Decreasing response to treatments • Choice of no further active treatment • Weight loss >10% in 6 months • Repeated unplanned admissions • “Sentinel event,” eg, serious fall, transfer to nursing home • Serum albumin <25 g/L • Eligible for DS1500 *	
COPD-specific indicators • FEV1 ≤30% • >3 hospitalizations in 12 months • LTOT criteria fulfilled • MRC 4/5 • R heart failure • ≥6/52 oral steroids in the last 6 months • Combination of other factors (anorexia, NIV, ITU, resistant organisms)	

**Notes:**

* The DS1500 is a form, completed by a health care professional, which enables someone who is terminally ill to claim PIP, ESA or AA under what the Department of Work and Pensions calls “Special Rules.” A prognostic estimate does not have to be included on the form, but terminal illness is defined in Social Security legislation as a progressive disease where death can reasonably be expected within 6 months.

**Abbreviations:** AA, attendance allowance; ADLs, activities of daily living; ESA, Employment and Support Allowance; FEV1, forced expiratory volume in 1 second; GSF-PIG, Gold Standards Framework Prognostic Indicator Guidance; ITU, intensive care; LTOT, long-term oxygen therapy; MRC, Medical Research Council; NIV, non-invasive ventilation; PIP, Personal Independence Payment.

**Table S2:** RADPAC

Components of score	Means of classifying patients on the basis of score
1. Moderately disabled; dependent (Karnofsky ≤50%)	No specific score suggested. Indicators designed to structure conversation and prompt assessment of different domains, leading to identification of needs, and prompting some form of anticipatory or advance care planning
2. Weight loss (10% in 6 months)	
3. CCF	
4. Orthopnea	
5. Patient mentions “end-of-life approaching”	
6. Signs of serious dyspnea (eg, dyspnea when speaking and use of accessory muscles)	

**Note:** Data from Thoonsen et al.^2^

**Abbreviations:** CCF, congestive cardiac failure; RADPAC, RADboud Indicators of Palliative Care Needs.

**Table S3:** SPICT

Components of score	Means of classifying patients on the basis of score
General indicators • Unplanned hospital admissions • Performance status poor or deteriorating (eg, in bed ≥50% time) • Dependent on others for care • Significant weight loss in the last 3–6 months and/or low BMI • Persistent symptoms • Person or family ask for palliative care or focus on quality of life Respiratory-disease specific • “Severe chronic lung disease” • Breathless at rest or on minimal exertion • Needs LTOT • Has needed ventilation for respiratory failure or ventilation is contraindicated	No specific score suggested

**Note:** Data from Scottish Government. SPICT: Supportive and Palliative Indicators Tool. 2016.^1^

**Abbreviations:** BMI, body mass index; LTOT, long-term oxygen therapy; SPICT, Supportive and Palliative Care Indicators Tool.

**Table S4:** NECPAL tool

Components of score	Means of classifying patients on the basis of score
1. Surprise question	Surprise question with answer “no,” and at least one other question (2, 3 or 4) with answer “yes”
2. Choice, request or need: any request to limit treatment or for palliative care from patient, family or team members	
3. General indicators • Nutritional decline (weight loss >10% in 6 months or albumin <2.5 g/dL) • Functional decline (Karnofsky <50%, Barthel <25, ECOG >2) • Other markers of frailty (two of the following in the last 6 months: pressure ulcer stage III–IV, >1 systemic infection, persistent dysphagia, delirium, falls >2) • Emotional distress (numerical verbal scale or HADS) • Comorbidity (≥2 chronic diseases) • >1 admissions in 12 months or increased need for care (residential care or home care)	
4. Specific indicators (two or more) • Breathless at rest or on minimal exertion • Difficult physical or psychological symptoms • FEV1 <30% or VC <40% or DLCO <40% • Needs LTOT • Symptomatic heart failure • >3 admissions in 12 months due to COPD exacerbations	

**Note:** Data from Gómez-Batiste et al.^4^

**Abbreviations:** DLCO, diffusing capacity of the lungs for carbon monoxide; ECOG, Eastern Cooperative Oncology Group; FEV1, forced expiratory volume in 1 second; HADS, hospital anxiety and depression score; LTOT, long-term oxygen therapy; NECPAL, Necesidades Paliativas; VC, vital capacity.

References1Scottish GovernmentSPICT: Supportive and Palliative Indicators Tool2016Available from: http://www.gov.scot/resource/doc/924/0111396.pdfAccessed June 28, 20172ThoonsenBEngelsYvan RijswijkEEarly identification of palliative care patients in general practice: development of RADboud indicators for PAlliative Care Needs (RADPAC)Br J Gen Pract201262602e625e6312294758310.3399/bjgp12X654597PMC34266013MeyerPAManninoDMReddSCOlsonDRCharacteristics of adults dying with COPDChest20021226200320081247583910.1378/chest.122.6.20034Gómez-BatisteXMartínez-MuñozMBlayCIdentifying patients with chronic conditions in need of palliative care in the general population: development of the NECPAL tool and preliminary prevalence rates in CataloniaBMJ Support Palliat Care20133330030810.1136/bmjspcare-2012-000211246447485Gold Standards Framework [homepage on the Internet]National Gold Standards Framework for End of Life Care2017Available from: http://www.goldstandardsframework.org.uk/Accessed June 28, 2017

## Figures and Tables

**Table 1:** Predictors of mortality in stable COPD ≤12 months

Individual prognostic variables							
Study	Study design	COPD diagnosis	Age, mean (SD)	Male (%)	FEV1% predicted mean (SD)	Events/total	Main exposure(s) of interest
Braun et al^56^	Single-center longitudinal cohort study within RESTOR COPD rehabilitation program, WI, USA Case–control study within this	Unclear	Overall mean NR ≈63	72	972±84 mL, ≈36%	?/39; 1-year follow-up	Markers of nutritional depletion: triceps skin fold, mid-arm muscle circumference, weight, estimated daily nutrient intake from 3-day dietary record, basal energy expenditure estimated from Harris–Benedict equation
Fan et al^46^	Multisite, longitudinal cohort study within RCT. 17 centers (NETT, medical arm)	Bilateral emphysema on CT, FEV1 ≤45% predicted, TLCO ≥100% predicted, RV ≥150% predicted	66.1 (6.1)	61.2	26.75 (7.2)	45/604; 4-year follow-up	Depression: BDI – self-reported 21-item measure. Score: 0–3 for each question. There are 21 questions. If ≥10 consistent with mild-to- moderate depression Anxiety: STAI – self- reported 20-item scale. Score: 20–80. Higher score = higher anxiety
Man et al^47^	Multisite, longitudinal cohort study (Lung Health Study)	Post-BD FEV1 <90% but ≥55% predicted and FEV1/FVC <0.7	53 (7)	63	78 (9)	329/4,803; mean follow-up of 7.5 years	CRP (mg/L)
Mannino et al^57^	Multisite, longitudinal cohort study (Lung Health Study)	Post-BD FEV1 <90% but ≥55% predicted and FEV1/FVC <0.7	Mean NR	62.6	55%–90%	500/5,887; 5-year follow-up	Pre- and post- bronchodilator lung function
Meyer et al^58^	Cross-sectional survey (National Mortality Followback Survey, USA)	ICD-9 codes: 490, 491, 492, 496	Mean NR	50.1	NR	COPD: 1,279/225,400 Non-COPD: 11,524/1,894,500	COPD, smoking status, weight, history of asthma
Polkey et al^59^	Multisite, longitudinal cohort study. 46 centers, 12 countries (ECLIPSE)	Post-BD FEV1 <80% predicted and FEV1/FVC <0.7	63.3 (7.0)	65	49.1 (15.7)	94/1,847; 3-year follow-up	Δ6MWT – minimal clinically important difference (m)
Austin et al^31^	Multisite, longitudinal cohort study within EHR, ON, Canada	ICD-9 codes: 491, 492, 496 ICD-10 codes: J41, J42, J43, J44	66 (median)	49	NR	14,124/638,926 (but 50% validation); 1-year follow-up	Elixhauser Comorbidity Index, Charlson Comorbidity Index, John Hopkin’s Comorbidity Index
Boeck et al^60^	Longitudinal observational cohort	Smoking history, spirometry	67 (10)	70	49 (17)	54/460; 2-year follow-up	ADO, B-AE-D, updated BODE, DOSE
Marin et al^30^	Pooled individual patient data of observational longitudinal cohort studies	Spirometry	66.4 (9.7)	93.3	53.8 (19.4)	1,245/3,633; 10-year follow-up	ADO, BODE, BODEx, eBODE, DOSE, SAFE
Martinez et al^61^	Multisite, longitudinal cohort study within RCT. 17 centers (NETT, medical arm)	Bilateral emphysema on CT, FEV1 ≤45% predicted, TLCO ≥100% predicted, RV ≥150% predicted	66.1 (6.1)	61.2	26.75 (7.2)	203/610; 4.5-year follow-up	ΔmBODE

**Notes:** ADO: age, dyspnea and obstruction; B-AE-D: BMI (B), severe AECOPD frequency (AE), mMRC dyspnea severity (D); BODE: BMI, airflow Obstruction, Dyspnea, and Exercise; DOSE: dyspnea, obstruction, smoking, exacerbation. ? represents unknown event number.

**Abbreviations:** AECOPD, acute exacerbation of COPD; BD, bronchodilator; BDI, Beck Depression Inventory; BMI, body mass index; CRP, C-reactive protein; CT, computed tomography; eBODE, exacerbations BODE; BODEx, BODEexercise capacity; EHR, electronic health records; FEV1, forced expiratory volume in 1 second; FVC, forced vital capacity; ICD-9, International Classification of Disease, ninth edition; 6MWT, 6-minute walk test; mMRC, modified Medical Research Council; mBODE, modified BODE; NETT, National Emphysema Treatment Trial; NR, not reported; RCT, randomized controlled trial; RV, residual volume; SAFE, obstruction, exercise, quality of life and exacerbations; STAI, State Trait Anxiety Inventory; TLCO, gas transfer for carbon monoxide.

**Table 2:** Individual prognostic variables identified predictive of mortality ≤12 months in stable COPD

Individual prognostic variables						
Variables (reference group)	Study	Adjustments	Methods	Results (95% CI)	Other reported results (95% CI)	Comments
CRP (quintile 1)	Man et al^47^	Age, ethnicity, sex, BMI, biochemically validated smoking status (salivary cotinine), FEV1% predicted	Cox	Adjusted RR over entire follow-up (7.5 years): Quintile 2: 0.98 (0.65–1.68) Quintile 3: 1.14 (0.78–1.68) Quintile 4: 1.13 (0.77–1.65) Quintile 5: 1.79 (1.25–2.56) Multiple regression model for 1-year mortality only, “significant” results reported: Age: *P*=0.002 Race: *P*<0.001 BMI: *P*=0.005 CRP: *P*=0.003	C-statistic CRP quintile 0.69 (0.58–0.81) Compared to age quintiles 0.70 (0.57–0.82), FEV1 quintiles 0.65 (0.53–0.77) When age, ethnicity, BMI and CRP combined in model C-statistic 0.82. No 95% CI reported. No other details of this model reported	Trend for CRP quintile, *P*<0.001, but poor discrimination between categories. Absolute levels lack clinical relevance. Limited results of 1-year mortality model presented
BDI (<5)	Fan et al^46^	Age, sex, ethnicity, marital status, educational level, annual income, mBODE quintile, antidepressant use, Hb level, RV%, TLCO%, max CPET workload, difference in % emphysema, perfusion ratio, Charlson–Deyo comorbidity	Logistic regression	Unadjusted 1-year mortality: BDI <10: 5.8% BDI ≥10: 10%, *P*=0.05 Adjusted OR: 5–7: 1.62 (0.54–4.85) 8–10: 1.59 (0.53–5.06) 11–14: 1.69 (0.56–5.06) ≥15: 1.88 (0.62–5.74)	No association between depressive symptoms and mortality when BDI analyzed as quintiles No effect modification between sex and depressive symptoms	No significant associations found
STAI (state or trait)	Fan et al^46^	Univariate analysis only presented	Logistic regression	No association found STAI state per 5-point change OR 0.96, *P*=0.6 STAI trait per 5-point OR 1.00, *P*=0.97 No adjusted analysis presented	None	No significant associations found
Smoking status (never smoker)	Meyer et al^58^	Age group, sex		Current smoker: OR 6.5 (4.3–9.9) Former smoker: OR 3.7 (2.5–5.3)		
Weight (overweight)	Meyer et al^58^	Age group, sex		Underweight: OR 4.5 (2.8–7.2) Correct weight: OR 1.6 (1.1–2.2)		
History of asthma (no history of asthma)	Meyer et al^58^	Age group, sex		OR 5.0 (3.2–7.8)		
Markers of nutritional depletion	Braun et al^56^	Age, sex (matching)	Group means only	Unable to extract any meaningful results		Methods and reporting inadequate

**Longitudinal measurement of individual prognostic variables**						

Change in mBODE over 6 months	Martinez et al^61^	Sex, ethnicity, baseline age, baseline mBODE		Decrease: ≥1 point decreased mortality risk: HR 0.57 (0.41–0.78, *P*<0.001) Increase: ≥1 point increased mortality risk: HR 2.35 (1.71–3.23, *P*<0.001)	C-statistic mBODE 0.68 Compared to FEV1 0.62, 6MWD 0.64, UCSD SOBQ 0.64. No 95% CI reported	Although multivariate model, “change” in score treated as individual prognostic variable
6MWD reduction 30 m over 12 months	Polkey et al^59^	None (accuracy not improved when % predicted used)		HR 1.93 (1.29–2.90, *P*=0.001)		

**Note:** BODE: BMI, airflow Obstruction, Dyspnea, and Exercise.

**Abbreviations:** BDI, Beck Depression Inventory; BMI, body mass index; CI, confidence interval; CPET, cardiopulmonary exercise testing; CRP, C-reactive protein; FEV1, forced expiratory volume in 1 second; Hb, hemoglobin; HR, hazard ratio; 6MWD, 6-minute walk distance; mBODE, modified BODE; OR, odds ratio; RR, risk ratio; RV, residual volume; STAI, State Trait Anxiety Inventory; TLCO, gas transfer for carbon monoxide; UCSD SOBQ, University of California San Diego shortness of breath questionnaire.

**Table 3:** Multivariable prognostic indices identified predicting mortality ≤12 months in stable COPD

Index	Study	Derivation/validation	Population	n events/n total	Prediction (months)	Discrimination	Calibration (plot)	Calibration (HL test)
ADO (10 points)	Boeck et al^60^	V	PROMISE (11 European tertiary centers)	?/530	12	0.72 (0.62–0.82)	NR	0.3
	Marin et al^30^	V	COCOMICS (11 Spanish cohorts)	131/3,633	6	0.701	NR	NR
	Marin et al^30^	V	COCOMICS (11 Spanish cohorts)	230/3,633	12	0.701	NR	NR
B-AE-D (simple) (6 points)	Boeck et al^60^	D	PROMISE (11 European tertiary centers)	?/530	12	0.78 (0.68–0.87)	NR	0.4
	Boeck et al^60^	V	COCOMICS (7 Spanish cohorts)	?/2,153	12	0.68 (0.63–0.72)	NR	0.5
	Boeck et al^60^	V	COMIC (single center, the Netherlands)	?/675	12	0.74 (0.65–0.83)	NR	0.2
B-AE-D (optimized) (26 points)	Boeck et al^60^	D	PROMISE (11 European tertiary centers)	?/530	12			
	Boeck et al^60^	V	COCOMICS (7 Spanish cohorts)	?/2,153	12			
BODE (10 points), four risk groups	Boeck et al^60^	V	PROMISE (11 European tertiary centers)	?/530	12	0.76 (0.65–0.87)	NR	0.9
	Marin et al^30^	V	COCOMICS (11 Spanish cohorts)	131/3,633	6	0.68	NR	NR
	Marin et al^30^	V	COCOMICS (11 Spanish cohorts)	230/3,633	12	0.682	NR	NR
Updated BODE BODEx (9 points), four risk groups	Boeck et al^60^	V	PROMISE (11 European tertiary centers)	?/530	12	0.78 (0.67–0.89)	NR	0.7
	Marin et al^30^	V	COCOMICS (11 Spanish cohorts)	131/3,633	6	0.651	NR	NR
	Marin et al^30^	V	COCOMICS (11 Spanish cohorts)	230/3,633	12	0.651	NR	NR
eBODE (12 points), four risk groups	Marin et al^30^	V	COCOMICS (11 Spanish cohorts)	131/3,633	6	0.68	NR	NR
	Marin et al^30^	V	COCOMICS (11 Spanish cohorts)	230/3,633	12	0.683	NR	NR
Comorbidity (Charlson)	Austin et al^31^	V	Canadian EHR	?	12			NR
Comorbidity (Elixhauser)	Austin et al^31^	V	Canadian EHR	?	12			NR
Comorbidity (John Hopkins)	Austin et al^31^	V	Canadian EHR	?	12			NR
DOSE (8 points), two risk groups	Boeck et al^60^	V	PROMISE (11 European tertiary centers)	?/530	12	0.64 (0.54–0.73)	NR	0.9
	Marin et al^30^	V	COCOMICS (11 Spanish cohorts)	131/3,633	6	0.632	NR	NR
	Marin et al^30^	V	COCOMICS (11 Spanish cohorts)	230/3,633	12	0.631	NR	NR
SAFE (9 points), four risk groups	Marin et al^30^	V	COCOMICS (11 Spanish cohorts)	131/3,633	6			
	Marin et al^30^	V	COCOMICS (11 Spanish cohorts)	230/3,633	12	0.641	NR	NR

**Notes:** ADO: age, dyspnea and obstruction; B-AE-D: BMI (B), severe AECOPD frequency (AE), mMRC dyspnea severity (D); BODE: BMI, airflow Obstruction, Dyspnea, and Exercise; DOSE: dyspnea, obstruction, smoking, exacerbation. ? represents unknown event number.

**Abbreviations:** AECOPD, acute exacerbation of COPD; BMI, body mass index; BODEx, BODE exercise capacity; eBODE, exacerbations BODE; EHR, electronic health records; HL, Hosmer-Lemeshow; mMRC, modified Medical Research Council; NR, not reported; SAFE, obstruction, exercise, quality of life and exacerbations.

**Table 4:** Shared variables across multivariable indices predicting mortality in COPD ≤12 months

Prognostic index	Demographic		Physiological		Exercise capacity	Patient reported		Prior history	
	Age	Smoking	FEV1% predicted	BMI	6MWT distance	Dyspnea*	Quality of life SGRQ	Severe exacerbations¥	Comorbidities
ADO	✓		✓			✓			
B-AE-D				✓		✓		✓	
BODE			✓	✓	✓	✓			
BODEx			✓	✓		✓		✓	
eBODE			✓	✓	✓	✓		✓	
mBODE			✓	✓	✓	✓			
Elixhauser comorbidity									✓
Charlson comorbidity									✓
John Hopkin’s comorbidity									✓
DOSE		✓	✓			✓		✓	
SAFE			✓		✓		✓	✓	

**Notes:**

* Includes different dyspnea measures: mMRC, Fletcher, and SOBQ.

¥ Exacerbation history measured over variable time frames, either last 12 or 24 months. ADO: age, dyspnea, and obstruction; B-AE-D: BMI (B), severe AECOPD frequency (AE), mMRC dyspnea severity (D); BODE: BMI, airflow Obstruction, Dyspnea, and Exercise; DOSE: dyspnea, obstruction, smoking, exacerbation.

**Abbreviations:** AECOPD, acute exacerbation of COPD; BMI, body mass index; BODEx, BODE exercise capacity; FEV1, forced expiratory volume in 1 second; 6MWT, 6-minute walk test; eBODE, exacerbations BODE; mBODE, modified BODE; mMRC, modified Medical Research Council; SAFE, obstruction, exercise, quality of life and exacerbations; SGRQ, St George’s Respiratory Questionnaire; SOBQ, shortness of breath questionnaire.

**Table 5:** Risk of bias assessment (included studies)

Study	Participation and attrition	Prognostic factor measurement	Outcome measurement	Confounding measurement	Analysis and reporting	Overall
Braun et al^56^	Inadequately described source and sample population. Small sample size	Reliance on recall. Missing data and blinding NR. Probable complete case analysis	Little detail reported. Inadequate events for number of variables explored	No comorbidities recorded. Unclear how confounders other than age and sex accounted for	Insufficient data reported. Inadequate statistical approach. Unable to extract relevant data	Quality poor. High risk of bias
Fan et al^46^	Adequately described but highly selected population, not representative of general COPD population	Measurement appears fine. Missing data and blinding NR. Probable complete case analysis	Measurement reliable. But inadequate events for number of variables explored	Well recorded, and many potential confounders included in analysis	Analysis and reporting fine. Likely underpowered for this question	Quality fine. Moderate-risk bias
Man et al^47^	Young cohort, mild disease, few comorbidities (excluded) so not very representative of general COPD population	Measurement appears fine. Missing data and blinding NR. Probable complete case analysis	Measurement reliable. Investigators limited number of covariates, but still danger of inadequate events for number of variables explored, particularly as quintiles = additional parameters estimated	Relevant confounders recorded and adjusted for in model(s)	Some aspects of modeling unclear. Stepwise (backward/forward) variable selection on the basis of *P*<0.2 univariate, *P*<0.1 multivariate. Log transformation of CRP for some analyses. Little reported on 1-year mortality model – *P*-values of “significant” results only and C-statistic no CIs	Quality moderate. Moderate-risk bias
Mannino et al^57^	Source population is adequately described but not a true population- based study. A clinical intervention trial that targeted early COPD	Used pre- and post-bronchodilator lung function data to determine whether these measures differ in ability to predict mortality	Outcome was all-cause mortality. Measurement reliable. Details on cause of death lacking in results data	Adjusted for age, sex race, smoking status, educational level, BMI and randomization, but other confounders such as comorbidities not adjusted for	Analysis and reporting fine	Moderate risk of bias
Meyer et al^58^	Limited detail given but fine. Concern regarding reliance on death certificate documentation of COPD as this is known to be inaccurate (both as cause of death and any mention of COPD) – misclassification risk		Death with COPD listed on death certificate ICD-9 codes. Not blinded since relatives asked about smoking and asthma history after death had occurred	Age and sex. No treatments recorded. No missing confounder data		Moderate risk of bias
Polkey et al^59^	Described in original study protocol fully, and briefly here	No missing data stated (those without two measurements excluded from analysis – no imputation attempted). Blinded for outcome as measured in advance. Do not appear to be blinded to each other, but only really one PF of interest and others covariates	All-cause mortality. First hospitalization. Composite of both. Clear definition of exacerbation/hospitalization too	Treatment not reported, only cardiovascular comorbidity reported. No comments re: missing data. Is a complete case analysis as those without paired 6MWD not included. But data presented both for analyzed group and whole group and no significant difference so bias unlikely. No – results not stratified or adjusted. Hazard ratio reliable	Yes – although no adjustments	Moderate risk of bias

**Multivariable prognostic indices**						

**Study**	**Participation and attrition**	**Prognostic factor measurement**	**Outcome measurement**	**Confounding measurement**	**Analysis and reporting**	**Overall**

Austin et al^31^	Likely representative. Health care databases so includes all severities, those with comorbidities, women represented. Relies on ICD codes – but previously found to be relatively sensitive/specific. Risk of misclassification. Little detail given about exclusions	Comorbidity data relied on databases of hospital discharges and other health care encounters, including billing claims. Possible misclassification bias dependent on quality of coding	All-cause mortality: occurrence of death within 365 days of index date. Fine but some could have been missed due to delays in recording, etc. Probably not significant in this large a population	Age and sex. Did not have data on BODE components	No CIs on C-statistics presented. No B coefficients presented. Unclear how comorbidities have been weighted	Moderate risk
Boeck et al^60^	Described in original papers. Some details given in this article. PROMISE cohort not representative – tertiary referral centers. Validation cohorts more varied but most secondary/tertiary care. Some differences between participants and dropouts (more severe?)	Self-reported number of exacerbations and severe exacerbations – reliance on recall. Ninety-eight patients excluded due to missing data (some due to missing data at baseline), plus 45 excluded due to no 1-year follow-up. Participants also excluded from validation cohorts if missing data – no attempts at statistical methods, eg, multiple imputation	Limited information provided but physician defined cause of death so some clinical ± record check implied	Ethnicity and treatments not recorded. But models tested adding in age, sex, FEV1%, smoking status, 6MWD and made little difference to discrimination. Those with missing data excluded. No imputation. Complete case analysis used – inefficient. Potential bias	Missing details in choice of variables – clinical vs statistical methods. Unclear basis on which decisions made. Model building strategy unclear. Seems to be based on clinical conceptual framework, but then Lasso mentioned, but results not reported and unclear whether used	Moderate risk
Marin et al^30^	Predominantly male. All under secondary/tertiary care. Adequate description of key factors in individual cohorts and overall. Women significantly underrepresented. Inadequate info on loss to follow-up provided but comment that very little long-term follow-up in these cohorts	All standard protocols. Limited reliance on recall (required for exacerbations but nothing else and likely to be well remembered as severe only). Cut points driven by indices tested. Blinded for outcome. Not blinded for each other as far as can tell	Different cohorts but probably fine	Not necessarily included in analysis – ie, treatments not included. Age a tricky one – included for ADO and in further analysis BODE stratified for age but this was not reported. Difficult to comment due to study design. In some ways does not matter as finding simple model to explain variance. If good discrimination not such an issue which factors/confounders included	Some results thoroughly presented, but some analyses missing, ie, stratification for age – very relevant. Some indices results not reported, eg, mBODE, CPI, SAFE, HADO, COPDSS, TARDIS. But in the Supplementary materials, some variables not available so not analyzed	Low risk
Martinez et al^61^	Yes described. Representative of this cohort, but not of general COPD population – highly selected. Fine methodologically but not representative of COPD population	Yes – factors measured. Cut points based on previous work	Death – little detail provided on how they obtained these data. But cohort, so likely fine	Treatment not included as covariate, but “maximal medical treatment” implies triple inhaled therapies, etc. Sex, ethnicity, age and baseline mBODE adjusted for in model Overall: no concerns	Clinically based model, no issues	Low risk

**Notes:** ADO: age, dyspnea and obstruction; BODE: BMI, airflow Obstruction, Dyspnea, and Exercise.

**Abbreviations:** BMI, body mass index; CI, confidence interval; COPDSS, COPD severity score; CPI, COPD Prognostic Index; CRP, C-reactive protein; FEV1, forced expiratory volume in 1 second; HADO, health, activity, dyspnea, obstruction; ICD-9, International Classification of Disease, ninth edition; Lasso, least absolute shrinkage and selection operator; 6MWD, 6-minute walk distance; mBODE, modified BODE; NR, not reported; PF, prognostic factor; SAFE, obstruction, exercise, quality of life and exacerbations; TARDIS, Tayside Allergy and Respiratory Disease Information System.
